# Expression of E-prostanoid receptors in nasal polyp tissues of smoking and nonsmoking patients with chronic rhinosinusitis

**DOI:** 10.1371/journal.pone.0200989

**Published:** 2018-07-24

**Authors:** Li Xie, Ai-Guo Liu, Li-Yan Peng, Su-Jie Wang, Yin-Ping Zhang, Xian-Song Wang

**Affiliations:** 1 Department of Otolaryngology-Head and Neck Surgery, Tongji Hospital, Tongji Medical College, Huazhong University of Science and Technology, Wuhan, P.R. China; 2 Department of Otolaryngology-Head and Neck Surgery, The Fifth Affiliated Hospital of Zheng Zhou University, Zhengzhou, Henan, P.R. China; 3 Department of Pathology, Tongji Medical College, Huazhong University of Science and Technology, Wuhan, P.R. China; Mie Daigaku, JAPAN

## Abstract

**Background:**

Different inflammatory reactions have been observed in the polyp tissues of nonsmokers and smokers with chronic rhinosinusitis (CRS). E-prostanoid (EP) receptors play a role in the inflammatory processes. Cigarette smoke (CS) exposure regulates EP-receptor expression levels promoting inflammatory mediator release from various inflammatory cells. In this study, we characterize the EP-receptor expression profiles in the polyps of nonsmoking and smoking CRS patients to explore the possible role of CS in the pathogenesis of chronic rhinosinusitis with nasal polyps (CRSwNP).

**Methods:**

Polyp biopsies were obtained from 28 non-smoking and 21 smoking CRSwNP patients. Histopathological characteristics were observed under a light microscope. The prostaglandin E_2_ (PGE_2_), TNF-α, and IL-8 contents in polyp tissues were detected using enzyme-linked immunosorbent assay. Immunostaining was used to locate EP receptors in polyps. Messenger RNA and protein expression of EP receptors were examined using quantitative real-time polymerase chain reaction and Western blot, respectively.

**Results:**

More severe inflammatory reactions occurred in polyp tissues of smoking CRSwNP patients. The PGE_2_, TNF-α, and IL-8 in tissue homogenate levels were significantly higher in smoking CRSwNP patients than those in nonsmoking CRSwNP patients. Moreover, the distribution of each EP receptor subtype was similar in both groups. Compared with the EP-receptor expression in nonsmokers, messenger RNA and protein of EP2 and EP4 receptor were significantly down-expressed in smoking patients, but EP1 and EP3 receptors did not show significant differences.

**Conclusion:**

CS exposure downregulates the expression levels of EP2 and EP4 receptors and stimulates the production of PGE_2_ and the proinflammatory cytokine IL-8 and TNF-α in polyp tissues of CRS patients. The down-expressed EP2 and EP4 receptors might be associated with severe inflammatory reactions in smoking CRSwNP patients.

## Introduction

Chronic rhinosinusitis (CRS) is characterized by chronic and persistent inflammation of nasal and paranasal sinus mucosa. Epidemiological analyses indicate that 20% of CRS patients exhibit nasal polyps [1, 2]. Clinically, CRS with nasal polyps (CRSwNP) is more recalcitrant and refractory than CRS without nasal polyps. Thus, its mechanism has become a significant interest among many clinicians and researchers over the last decade [3]. Current studies suggest that the development of CRSwNP might involve a complex of intrinsic and exogenous factors, including infections, allergy, environmental exposure, and genetic predisposition [4].

Cigarette smoke (CS) exposure is an important environmental factor. A higher CRSwNP prevalence and a less favorable response to sinus surgery have been reported in cigarette smokers compared with nonsmokers [5–7]. Moreover, chronic exposure to CS aggravates eosinophilic inflammation, which favors nasal polyp formation [8]. These results suggest that CS might play a role in CRSwNP pathogenesis. In the lower airway, CS exposure can damage epithelial cells and promote the release of various proinflammatory mediators and inflammatory cell recruitments, thereby leading to numerous inflammatory diseases, such as asthma, emphysema, and chronic obstructive pulmonary diseases [9, 10]. Although these proinflammatory mediators and infiltrating cells are also associated with the development of nasal polyps in CRS patients [4], relatively little is understood whether smoking also promotes cell recruitment or causes a change in relevant mediator profiles in nasal polyps to participate in the formation of nasal polyps or CRSwNP.

Prostaglandin E_2_ (PGE_2_) is a one of the most important arachidonic acid metabolites. In inflammatory processes, PGE_2_ is usually produced in high amounts and promotes or inhibits inflammatory responses by regulating the activities of various inflammatory cells or modulating immune cell functions [11]. The contradictory actions of PGE_2_ are mediated by binding four diverse E-prostanoid (EP) receptors, namely, EP1, EP2, EP3, and EP4 receptors. EP1/EP3 receptors mediate the proinflammatory effects, whereas EP2/EP4 receptors mediate the anti-inflammatory effects [12]. Several studies showed that CS influences the expression of EP receptors in inflammatory cells to generate proinflammatory effects [13]. Whether CS exposure can change EP receptor expression levels in nasal polyp tissues of CRSwNP patients to affect the effects of PGE_2_ remains to be clarified. In this study, we compared the characteristics of EP receptor expression in polyp tissues between the nonsmoking and smoking CRSwNP patients to explore the possible role of cigarette smoking in the pathogenesis of CRSwNP.

## Methods

### Subjects

A total of 49 patients with CRSwNP were enrolled from March 2016 to January 2017 at the Department of Otolaryngology-Head and Neck Surgery, Tongji Hospital (Wuhan, China). CRSwNP was diagnosed using the typical symptoms (nasal obstruction, nasal discharge, facial pain, reduction, and loss of smell) that persisted beyond 12 weeks, endoscopic signs of nasal polyps, and a positive computed tomography scan change according to the current European Position Paper on Rhinosinusitis and Nasal Polyps 2012. CRSwNP was classified as eosinophilic when percent tissue eosinophils exceeded 10% of total inflammatory cells [14]. Up to 28 subjects were nonsmoking patients with CRSwNP who neither smoke nor live or work in a smoky environment. A total of 21 patients were currently active smokers with CRSwNP. Each smoking patient smoked more than 20 cigarettes per day for the last five years or more [15]. According to the criteria, smoking volume was defined in pack-years (number of packs smoked per day times the number of years of smoking) [16]. Subjective symptoms were scored on a visual analog scale (VAS) as mentioned elsewhere [17]. This focused on five major symptoms including rhinorrhea, nasal obstruction, decreased sense of smell, facial pain or fullness, and headache. A total symptom score was calculated based on the sum of these five VAS symptom domains. Endoscopy findings were recorded using Lund-Kennedy scoring system [18]. Sinus computed tomography (CT) findings were staged according to the Lund-Mackay system [19]. Clinical information of subjects is described in Table 1.

**Table 1:** Patients’ clinical data.

Characteristic	N-NP	S-NP	*P* value
Subject, n	28	21	-
Female sex, n (%)	10(35.7%)	6(28.6%)	NS
Age(years), median(IQR)	37.5 (22.5–48.0)	38.5(20.0–54.0)	NS
Eosinophilic CRSwNP, n (%)	12(42.9%)	9(42.9%)	NS
Smoking volume, median(IQR)	-	22.0 (7.5–49.0)	-
Total symptom scores, median (IQR)	18(13.0–23.0)	19(15.0–25.0)	NS
CT scores, median (IQR)	12(8.3–16)	15(13–19)	0.037
Endoscopy scores, median (IQR)	6(4–7)	9(5.5–11)	0.041

N-NP, nonsmoking CRSwNP; S-NP, smoking CRSwNP; NS, not significant.

All subjects were excluded from this study if they had a diagnosis of atopy, aspirin hypersensitivity, asthma, antrochoanal polyps, cystic fibrosis, or primary ciliary dyskinesia. The use of any topical or systematic medications, including steroids, nonsteroidal anti-inflammatory drugs, and anti-leukotrienes that may affect the research measurements, was prohibited in all patients for at least four weeks before surgery. This study was approved by the ethics committee of Tongji Hospital of Huazhong University of Science and Technology and conducted with written informed consent from all patients.

### Polyp biopsies

Tissues from the apical region of polyps were obtained during functional endoscopic sinus surgery. Fresh nasal biopsies were divided into at least two parts. One part was fixed in 10% formaldehyde and embedded in paraffin for hematoxylin–eosin (HE) and immunohistochemical staining. The other parts were snap frozen in liquid nitrogen and then stored at −80 °C for RNA and protein preparation.

### Histopathological examination

Paraffin sections were stained with HE and observed with a Nikon microscope. The number of inflammatory cells (eosinophils, neutrophils, and mononuclear cells) and mucosal glands in lamina propria was counted at 10 random fields of high-power (HP) magnification (×400). The results were expressed as cell or gland counts per HP field. Basement membrane thickness (BMT) and sub-epithelial edema were scored based on degree (0, none; 1, mild; 2, moderate; or 3, marked) at a magnification of ×100, as described in a previous study [20]. Collagen deposition was evaluated by Masson staining. The percentage of total collagen content was quantified using Image pro-plus 6.0 (Media Cybernetics, Inc., MD, USA) [21]. According to the criteria similar to that of Gao *et al* [22], squamous metaplasia was identified from specimens, in which the pseudostratified columnar epithelium was replaced by stratified squamous epithelium with total loss of cilia. Goblet cells were stained with Alcian blue and counted per millimeter of intact epithelium. Histological analysis was conducted by two pathologists who were blind to clinical data, as previously described [23].

### Measurements of PGE_2_, TNF-α, and IL-8 in polyp tissues

Each 0.1 g of tissue specimens was homogenized in 1 mL of buffer (0.1 M phosphate, pH 7.4, containing 1 mM EDTA). The homogenates were sonicated and centrifuged at 3000 rpm for 10 min at 4 °C. The contents of PGE_2_, TNF-α, and IL-8 in supernatants were measured using enzyme-linked immunosorbent assay kits according to manufacturer’s protocols. PGE_2_ kits were from Cayman, Michigan, USA. TNF-α and IL-8 kits were from R&D Systems, MN, USA. The detection limit of the assay was 15 pg/ml for PGE_2_, 4 pg/ml for IL-8 and TNF-α.

### Immunohistochemistry

Paraffin sections were used for immunohistochemical staining as described previously [23]. Briefly, sections were incubated with 3% hydrogen peroxidase to block endogenous peroxidase activity, with 5% bovine serum albumin to block nonspecific binding sites. Sections were incubated with polyclonal rabbit antihuman EP receptors (1:400 dilution for EP1, EP2, or EP3 and 1:200 for EP4, Cayman Chemicals, Michigan, USA) or polyclonal rabbit antihuman myeloperoxidase (MPO) (1:100 dilution, ZSGB-BIO, Beijing, China) overnight at 4 °C. The primary antibodies were detected with the StreptAvidin Biotin-peroxidase Complex kit (Boster Biotechnology, Wuhan, China). The immunoreaction was visualized using 3,3-diaminobenzidine-tetrahydrochloride, which causes brown staining of positive cells. Negative controls were performed by omitting primary antibodies and using non-immune sera of the same species.

### Quantitative real-time polymerase chain reaction (RT-PCR)

Total RNA from tissues was extracted and reverse transcribed to cDNA. Quantitative RT-PCR was performed using StepOnePlus system (ABI, Foster City, USA) and SYBR Premix Ex Taq kit (TaKaRa Biotechnology, Dalian, China) as described previously [23]. The PCR amplification consisted of one cycle at 95 °C for 2 min, followed by 40 cycles of denaturation at 95 °C for 10 s, annealing at 60 °C for 10 s, and then extension at 72 °C for 15 s. At the end of each PCR run, a melting curve analysis was used to confirm production specificity. Glyceraldehyde phosphate dehydrogenase (GAPDH) gene expression was used for normalization. Relative gene expression was calculated using comparative cycle threshold (2^−ΔΔ^Ct) method. No PCR product was amplified in the negative control. The following primers for PCR were adopted: EP1, forward 5'-GGTGTCGTGCATCTGCTGGA-3' and reverse 5'- CAAGAGGCGAAGCAGTTGGC-3' (187 bp); EP2, forward 5'-AGACGGACCAC CTCATTCTC-3' and reverse 5'-GATGGCAAAGACCCAAGG-3' (176 bp); EP3, forward 5'-CCCGCCTCAACCACTCCTA-3' and reverse 5'-CACCGATCCGCAAT CCTC-3' (107 bp); EP4, forward 5'-AACTTGATG GCTGCGAAGACCTAC-3' and reverse 5'-TTCTAATATCTGGGCCTCTGCTGTG-3' (128 bp); GAPDH, forward 5'- AGAAGGCTGGGGCTCATTTG -3' and reverse 5'-AGGGGCCATCCACAGTCT TC -3' (258 bp).

### Western blot

Total protein was extracted by homogenizing tissues in radioimmunoprecipitation assay/sodium lauryl sulfate and protease inhibitors as described previously [23]. Denatured total proteins (50 μg) were separated by sulfate-polyacrylamide gel electrophoresis followed by transferring into polyvinylidenedifluoride membranes. The membranes were incubated with block solution containing Tween-20 and 5% fat-free milk powder for 2 h at room temperature, and then incubated with polyclonal rabbit antihuman EP receptors (EP1, 1:1000 dilution; EP2, 1:1200 dilution; EP3, 1:1500 dilution and EP4, 1:500 dilution) overnight at 4 °C. Negative controls were performed in the absence of primary antibody or including an isotype control antibody. Membranes were incubated with horseradish-peroxidase conjugated goat anti-rabbit antibody (Sigma) for 1 h at room temperature. The immunoreactions were detected using a chemiluminescent method (Supersignal West Pico Chemiluminescent Substrate, Thermo, MA, USA) and exposed to GeneGnome HR Model (Synoptics Ltd., Cambridge, UK). The protein levels were semiquantified as ratios to the GAPDH band intensities using GeneTools.

### Statistical analysis

For continuous variables, the results were presented as medians and interquartile ranges or in box and whisker plots representing the medians and interquartile ranges. The nonparametric Kruskal-Wallis H test was used for data analysis within different groups. The Mann-Whitney U-test was applied for between-group comparisons. Difference in proportions was tested using the chi-square test or Fisher’s exact test. Significance was set to *p* < 0.05.

## Results

### Clinical data

In the present study, CRSwNP smokers and CRSwNP nonsmokers were comparable in terms of sex, age distribution and the proportions of eosinophilic CRSwNP. No significant difference in VAS symptom scores between the two groups was found. In comparison with non-smoking patients, smoker patients had significantly higher preoperative CT scores, and endoscopic scores (Table 1).

### Histopathological characteristics

**Fig 1:**
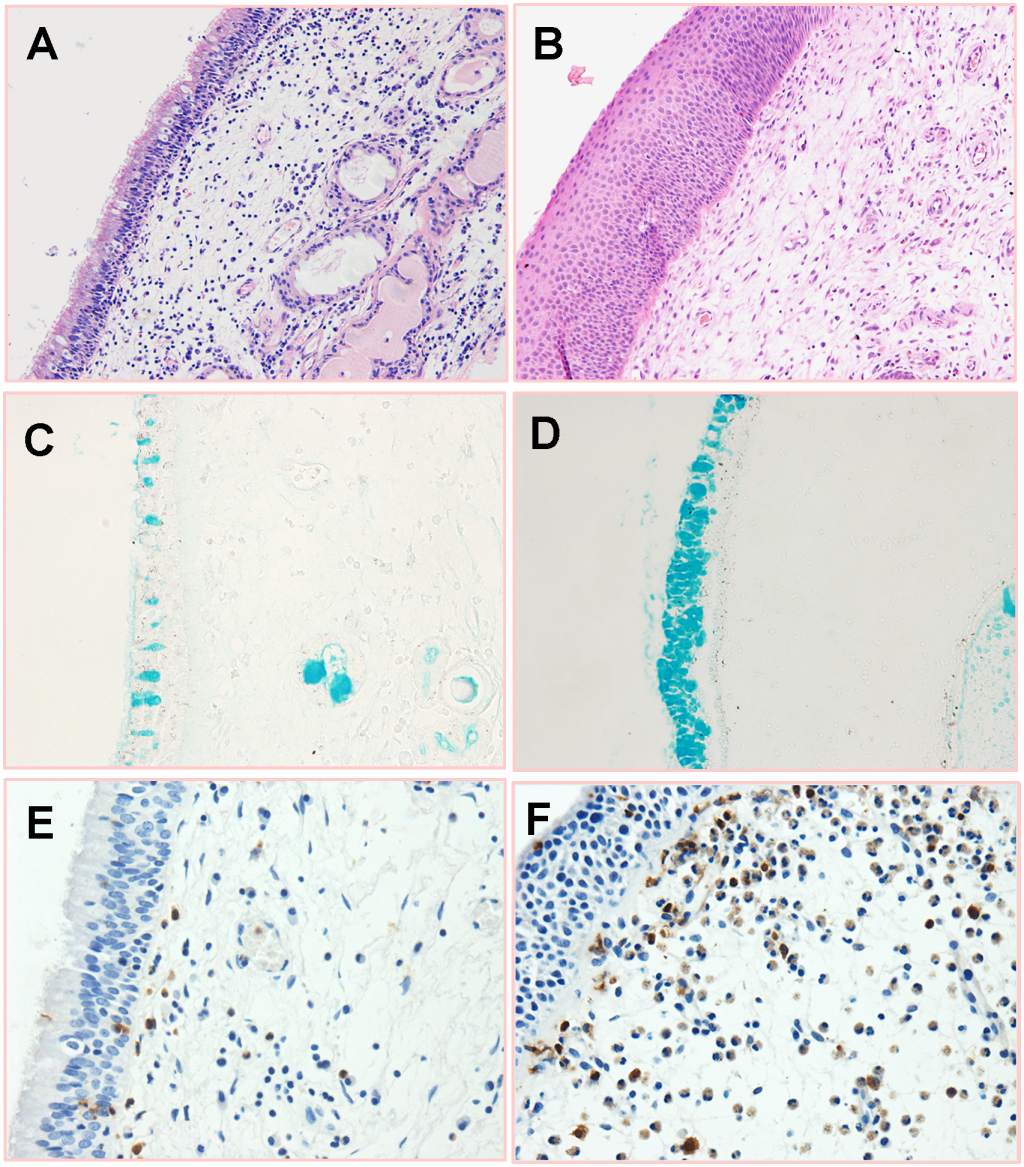
Representative photomicrographs of HE, Alcian blue or immunohistochemistry staining for polyp tissues. (A) Epithelium hyperplasia (HE, ×200). (B) Epithelial squamous metaplasia (HE, ×200). (C) Epithelial goblet cells in the nonsmoking CRSwNP patients (Alcian blue, ×400). (D) Goblet cell hyperplasia in the smoking CRSwNP patients (Alcian blue, ×400). E-F: Immunohistochemistry stainings for myeloperoxidase (MPO) in the CRSwNP nonsmokers (E) and CRSwNP smokers (F) (Original magnification, ×400).

Epithelium cell and goblet cell hyperplasia, basement membrane thickening, stromal edema, and inflammatory cell infiltration can be observed in smoking and nonsmoking CRSwNP patients (Fig 1). The quantitative analysis for the histopathological changes in polyp tissues from both patient groups is summarized in Table 2. Neutrophils (MPO+) were the prominent infiltrating inflammatory cell in nasal polyps of smokers and nonsmokers. The amounts of total inflammatory cells, neutrophils, and goblet cells were significantly higher in smoking CRSwNP patients than those in nonsmoking CRSwNP patients. Squamous metaplasia was found in 7/21 (33.3%) of nasal polyps in smoking CRSwNP patients and 2/28 (7.1%) in nonsmoking CRSwNP patients. The rate of squamous metaplasia was significantly higher in smoking CRSwNP patients compared with that in nonsmoking CRSwNP patients. No statistical differences in stromal edema, collagen deposition, BMT, and the count of eosinophils, mononuclear cells, and submucosal glands between two groups were observed.

**Table 2:** Histopathological features of polyp tissues from smoking CRSwNP and nonsmoking CRSwNP patients.

Characteristics	N-NP (n = 28)	S-NP (n = 21)	*P* values
Squamous metaplasia, n (%)	2(7.1%)	7(33.3%)	0.049
Goblet cells	19.0(14.7–28.7)	32.3(18.7–48.7)	0.020
BMT score	1.0(1.0–2.0)	2.0(1.0–2.0)	NS
Eosinophils	4.1(1.0–18.8)	5.5(3.8–39.0)	NS
Neutrophils (MPO+)	8.8 (6.2–14.0)	15.3(8.9–25.1)	0.01
Mononuclear cells	57.5 (36.3–78.5)	66.0 (51.5–86.5)	NS
Total inflammatory cells	75.0 (57.8–105.8)	99.0 (86.0–166.5)	0.042
Mucosal glands	1.7 (0.4–3.3)	2.0(0.9–3.2)	NS
Collagen deposition	8.4%(1.2%-16.8%)	11.1%(1.4%-22.1%)	NS
Subepithelial edema	1.8(1.0–2.0)	1.5(1.0–2.0)	NS

N-NP, nonsmoking CRSwNP; S-NP, smoking CRSwNP; BMT, basement membrane thickness; MPO, myeloperoxidase; NS, not significant.

### Levels of PGE2, TNF-α, and IL-8

**Fig 2:**
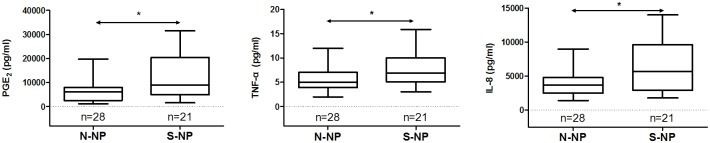
The contents of PGE2, TNF-α, and IL-8 in polyp tissue homogenates of the smoking CRSwNP and nonsmoking CRSwNP. Values are reported as medians and interquartile ranges. Mann-Whitney U test was applied for between-group comparisons. N-NP, nonsmoking CRSwNP; S-NP, smoking CRSwNP. *P < 0.05.

As shown in Fig 2, the values of PGE_2_, TNF-α, and IL-8 in the polyp tissues were significantly higher in smoking CRSwNP patients than those in nonsmoking CRSwNP patients.

### Immunohistochemical staining for EP receptor subtypes

**Fig 3:**
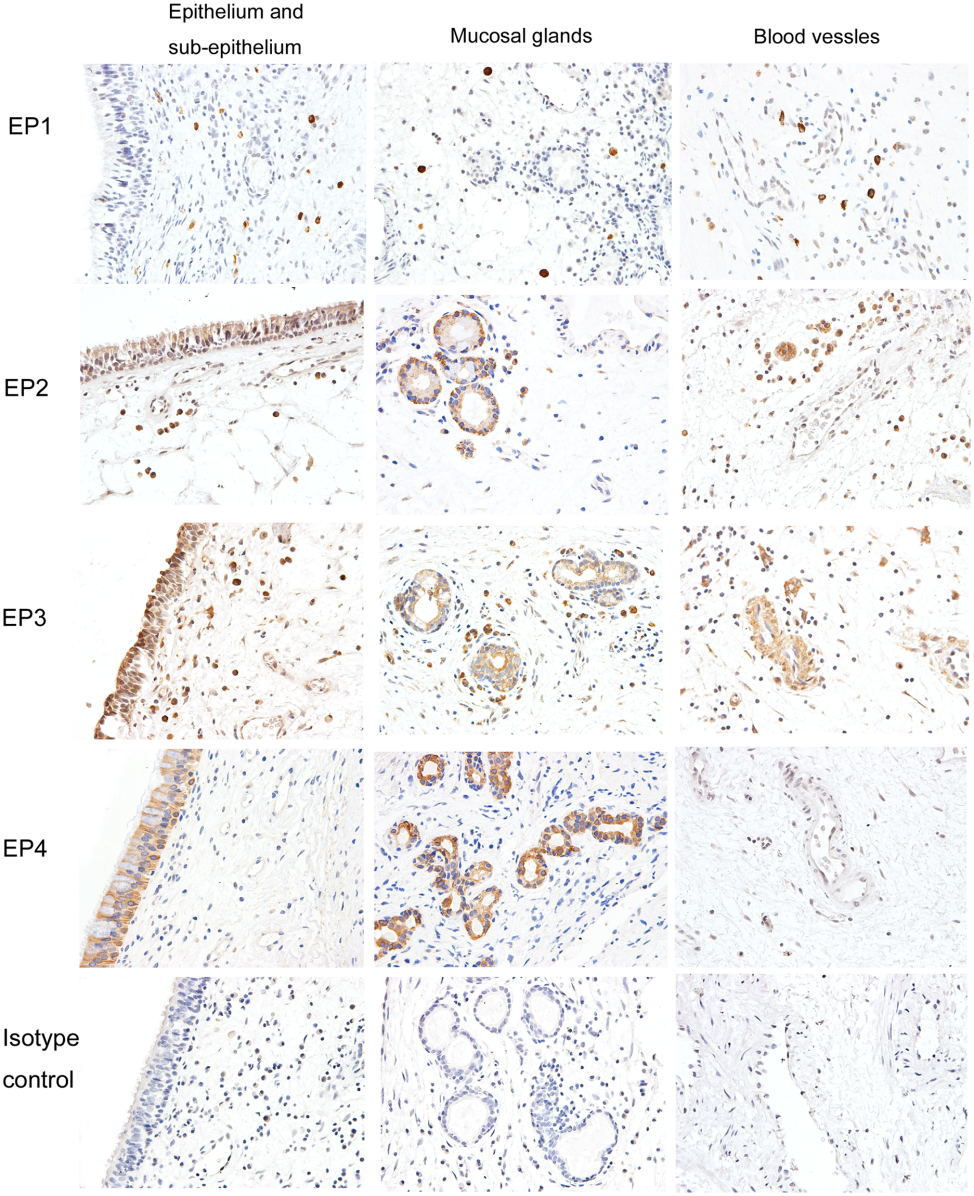
Representative immunostainings for EP receptors in CRSwNP smokers (Magnification, ×400). EP1 is expressed primarily on infiltrating inflammatory cells in lamina propria. Both EP2 and EP3 are expressed in inflammatory cells, mucosal epithelium, and glands. EP3 is also present in blood vessels. EP4 is located in the epithelium and glands.

Immunohistochemical staining for EP1, EP2, EP3, and EP4 receptors in nasal polyp tissues is shown in Fig 3 (CRSwNP smokers) and S1 Fig (CRSwNP nonsmokers). The cellular location of each EP receptor subtype was similar in smoking and nonsmoking CRSwNP patients. EP1 was expressed primarily on infiltrating inflammatory cells in sub-epithelial regions. Both EP2 and EP3 were expressed in inflammatory cells, mucosal epithelium, and glands; however, EP3 was expressed in blood vessels as well. Moreover, EP4 was expressed on the epithelium and glands.

### mRNA expression of EP receptor subtypes

**Fig 4:**
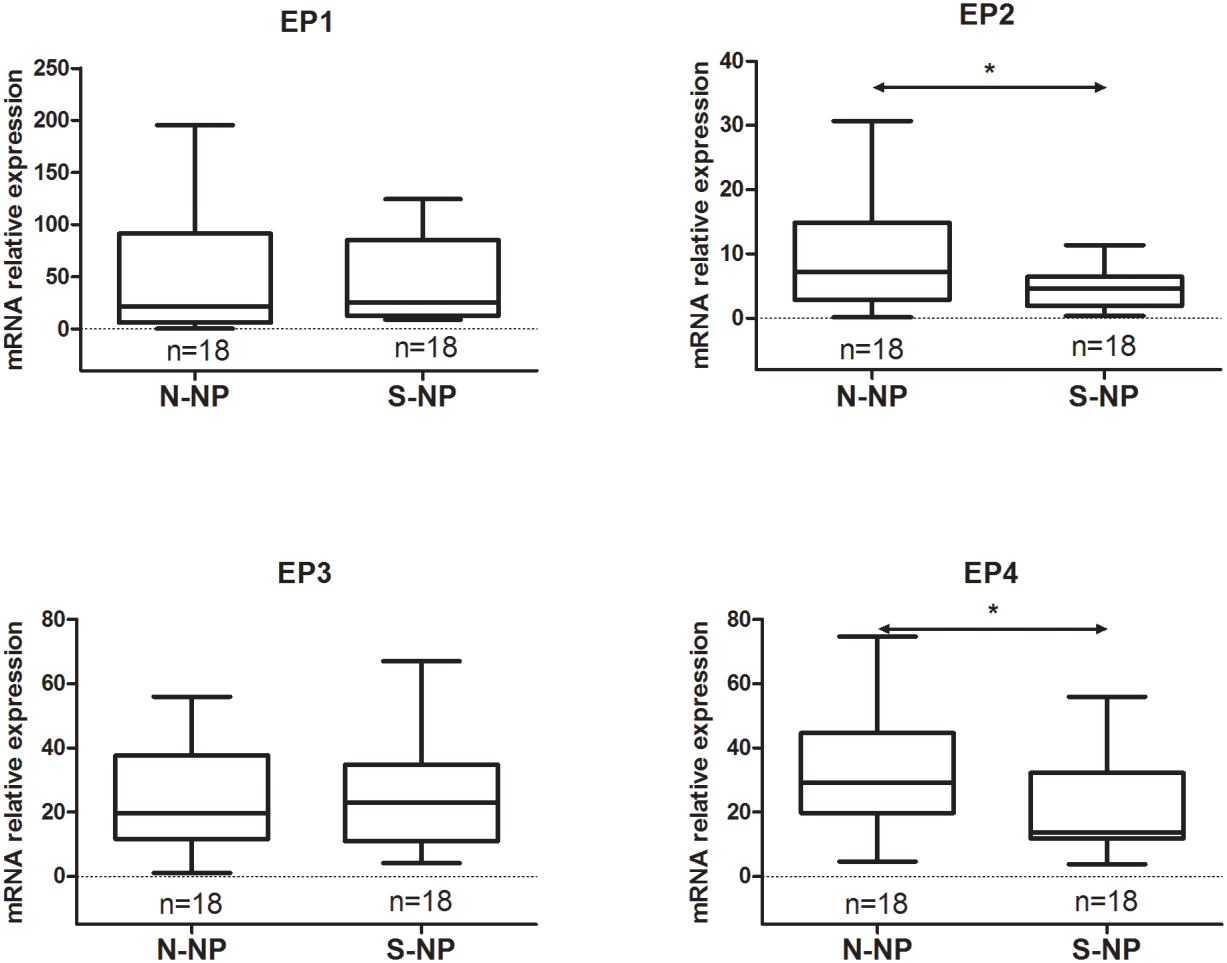
Comparison of EP receptor mRNA expression between the nonsmoking CRSwNP and smoking CRSwNP groups. N-NP, nonsmoking CRSwNP; S-NP, smoking CRSwNP. *P < 0.05.

Comparing the mRNA expression of the EP receptor subtype between the nonsmoking and smoking groups, as illustrated in Fig 4, EP2 or EP4 receptor was significantly downregulated in the smoking group compared with the nonsmoking group (*p* < 0.05). No significant differences were observed in the mRNA expression of EP1 or EP3 receptor between the two groups.

### Protein expression of EP receptor subtypes

**Fig 5:**
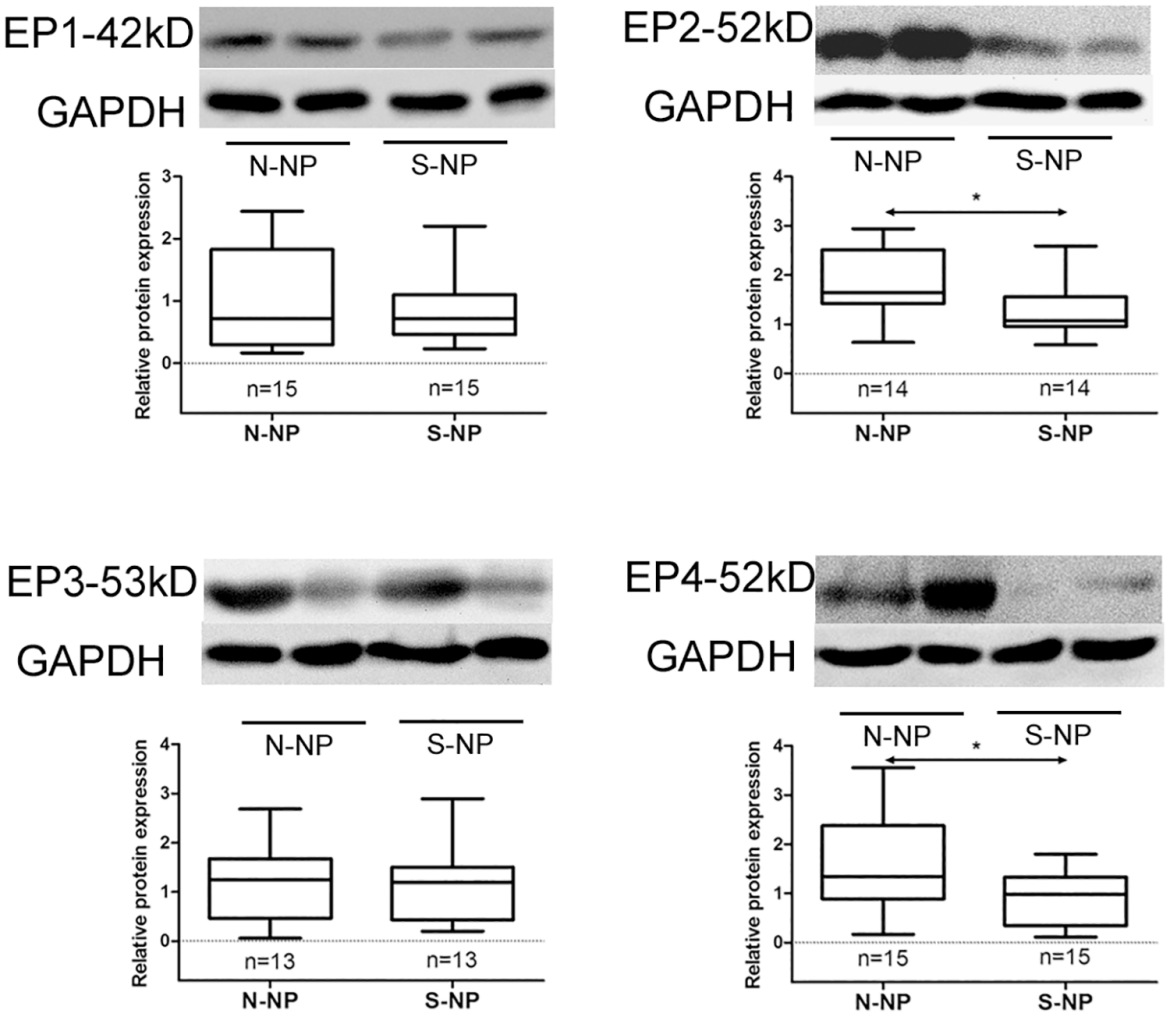
Comparison of EP receptor protein expression between the nonsmoking CRSwNP and smoking CRSwNP groups. Representative immunoblots are shown. N-NP, nonsmoking CRSwNP. S-NP, smoking CRSwNP. *P < 0.05.

The expression levels of the EP receptor proteins in polyp tissues from nonsmoking and smoking CRSwNP patients are illustrated in Fig 5. The EP2 and EP4 receptors were significantly downregulated in smokers compared with nonsmokers (*p* < 0.05). However, the protein levels of EP1 and EP3 receptors between the two groups showed no significant differences.

## Discussion

The reports about the clinical impact of smoking on CRSwNP patients have been discrepant. Zeynep found that there was no correlation between smoking and polyp size, CT scores, or subjective symptom scores [24]. Erbek [25] and Kilty *et al* [26] reported that smoking was not associated with polyp stage, CT score, but related to higher symptom scores. The present study showed that there were higher preoperative CT scores and endoscopic scores in smoking CRSwNP patients, which was consistent with those reported by Uhliarova *et al* [27]. Higher scores of CT and endoscopy imply more extensive inflammatory lesions present in smoking CRSwNP patients. Our study also demonstrated that CS exposure is associated with an increased CRS prevalence. The results suggest that CS exposure might be involved in the degree of nasal damage in CRS patients.

The histopathological changes of nasal mucosa in smokers have been reported to be different from those in nonsmokers [15]. Few studies indicated that CS, which is considered a potent proinflammatory stimulator in the lower airway, contributes to nasal polyp development [9, 28]. However, relevant reports about the effects of smoking on polyp histopathology of CRSwNP patients were controversial [22, 24, 27]. In this study, we found that the inflammatory reactions, including cellular infiltrates, edematous stroma and epithelial remodeling, which have been reported in previous studies [4, 21], were present in the polyp tissues of smoking and nonsmoking CRSwNP patients. Our finding that the epithelial squamous metaplasia and goblet cell hyperplasia were more prominent in smoking patients than in nonsmoking patients corresponds with the study of Gao *et al* [22] in Chinese patients, as well as in other studies performed in Asian populations [15]. The increased number of total inflammatory cells and neutrophils in the polyp tissues of smokers corresponds with the report that CS inhalation causes an increased numbers of macrophages, neutrophils, and T lymphocytes in bronchoalveolar lavage [10, 29]. The results suggest that CS exposure might be in favor of furthering the inflammatory reactions in the nasal polyp tissues.

Various proinflammatory factors, including cytokines, chemokines, and adhesion molecules, have been implicated in the development of nasal polyps [4]. IL-8, which is produced primarily from epithelial cells and macrophages, is increased in nasal polyps versus normal control mucosa [30]. IL-8 is a chemoattractant for neutrophils and elevated IL-8 level attracts more neutrophils to the nasal mucosa or polyp tissues. TNF-α is produced by macrophages, neutrophils, T-cells, mast cells, and epithelial cells. Moreover, TNF-α mRNA and protein expression levels are increased in nasal polyps versus inferior turbinate tissues [4]. TNF-α has been correlated with increased IL-8 expression and enhances neutrophil chemotaxis and migration [31]. TNF-α stimulates adhesion molecule expression in endothelial cells and upregulates CC chemokine ligand 2 in fibroblast cultures derived from nasal polyps, thereby facilitating the recruitment of inflammatory cells [32]. Studies showed that inflammatory cytokines, such as IL-1β, IL-6, IL-8, RANTES, and TNF-α, are significantly increased in the bronchoalveolar lavage of smokers [15]. In this study, the concentrations of IL-8 and TNF-α in polyp tissues from smoking CRSwNP patients were significantly higher compared with those in nonsmoking CRSwNP patients. The results suggest that CS might induce inflammatory cytokine release in polyps as it does in the lower airways. Moreover, high levels of IL-8 and TNF-α might account for the increased number of total inflammatory cells or neutrophils accumulating in the nasal polyps of smoking CRSwNP patients. Overproduction of IL-8 and TNF-α attracts more inflammatory cells into nasal polyp tissues. Conversely, the infiltrating cells release more mediators, including IL-8 and TNF-α after activation. Both of which lead to amplification of the inflammatory response and cause severe inflammatory injuries in the polyps of smoking CRSwNP patients.

PGE_2_ is one of the most abundant arachidonic acid metabolites in the body and is primarily produced by epithelial cells, endothelial cells, smooth muscles, and monocytes/macrophages in the airways, as well as by infiltrating cells, such as neutrophils, eosinophils, and mast cells, in inflammatory tissues [33]. Various proinflammatory and mitogenic stimuli, such as lipopolysaccharides, proinflammatory cytokines (TNF-α and IL-1β), or growth factors, have been proved to enhance of PGE_2_ synthesis and release; thus the PGE_2_ level is usually increased largely at inflammation sites [34, 35]. Several in vitro studies have reported that CS extract can induce PGE_2_ production by activating or upregulating the expression of three key enzymes, namely, phospholipase A2, cyclooxygenase-2, and membrane-bound prostaglandin E synthase-1, for catalyzing the PGE_2_ biosynthesis from arachidonic acid [13, 36, 37]. In this study, we found that PGE_2_ concentration in the nasal polyp tissues was statistically higher in smoking patients than in nonsmoking patients. The result suggests that CS might be a potential stimulator for PGE_2_ production. However, it is unclear whether the increased PGE_2_ production in smoker was attributed to the smoking-induced inflammatory response or the direct promotion of eicosanoid metabolism by enhancing the activities of key enzymes, or other unknown mechanism yet.

PGE_2_ is a known potent proinflammatory mediator involved in the initiation and progression of inflammation [12]. In addition to directly causing inflammatory response, PGE_2_ also plays a critical role in regulating the inflammatory process by modulating various immune cells [38]. PGE_2_ promotes the activation of T_H_17 cells to produce IL-17 or stimulate human T lymphocytes to produce IL-8 [39, 40]. IL-17 or IL-8 recruits more monocytes and/or neutrophils to the inflammation site and sustains inflammation. However, PGE_2_ also exerts an anti-inflammatory action by inhibiting the activities of various cells, such as neutrophils, monocytes, and epithelial cells [12]. The opposing effects of PGE_2_ in the inflammatory processes are performed by EP1, EP2, EP3, or EP4 receptors [41]. In general, EP2 and EP4 receptors mediate anti-inflammatory actions by activating adenylate cyclase to increase intracellular cAMP concentration [42–46]. By contrast, EP1 and EP3 receptors increase intracellular calcium (EP1) or decrease intracellular cAMP contents (EP3) to mediate proinflammatory effects [47–49]. Aside from the differential EP receptor subtype, the altered expression or coexpression of EP receptor in tissues causes diverse or contrasting PGE_2_ effects [50]. In the present study, EP2 and EP4 receptor expression levels were significantly lower in the smoking group compared with those in the nonsmoking group. This result suggests that smoking exposure might downregulate the expression levels of EP2 and EP4 receptors in nasal polyps. Moreover, the decreased expression levels of EP2 and EP4 receptors will weaken the anti-inflammatory effects of PGE_2_, thereby relatively enhancing the EP1 and EP3 receptor-mediated proinflammatory actions. Since both EP1 and EP3 receptors were primarily expressed in the infiltrating inflammatory cells, we speculate that the increased PGE_2_ level would promote the activities of inflammatory cells to release more inflammatory mediators by activating EP1/EP3 receptors, thereby leading to more serious inflammatory reactions in polyp tissues from the smoking CRS patients. The more intense inflammatory responses might contribute to the increased CT scores and endoscopic scores observed in smoking CRSwNP patients.

## Conclusions

In this study, we found that the cellular distribution of the four EP receptors was similar in the polyp tissues of smoking and nonsmoking CRSwNP patients. CS exposure enhanced PGE_2_ production and the proinflammatory cytokines IL-8 and TNF-α and downregulated the expression levels of EP2 and EP4 receptors in nasal polyps of CRS patients. The down-expressed EP2 and EP4 receptors might be associated with severe inflammatory reactions in smoking CRSwNP patients. These results suggested that CS exposure might play a role in the pathogenesis of CRSwNP by affecting EP receptor expression.

## Supporting information

S1 Fig Representative immunostainings for EP receptors in CRSwNP nonsmokers (Magnification, ×400). (TIF)

S1 Dataset Original data of this work. (XLSX)

S1 File Original immunoblots of EP receptor protein expression. (RAR)
