# Supplementary figures and images for: Expression of E-prostanoid receptors in nasal polyp tissues of smoking and nonsmoking patients with chronic rhinosinusitis
Source: PLoS One. 2018 Jul 24;13(7):e0200989. doi: 10.1371/journal.pone.0200989 (PMC6057645; doi:10.1371/journal.pone.0200989)

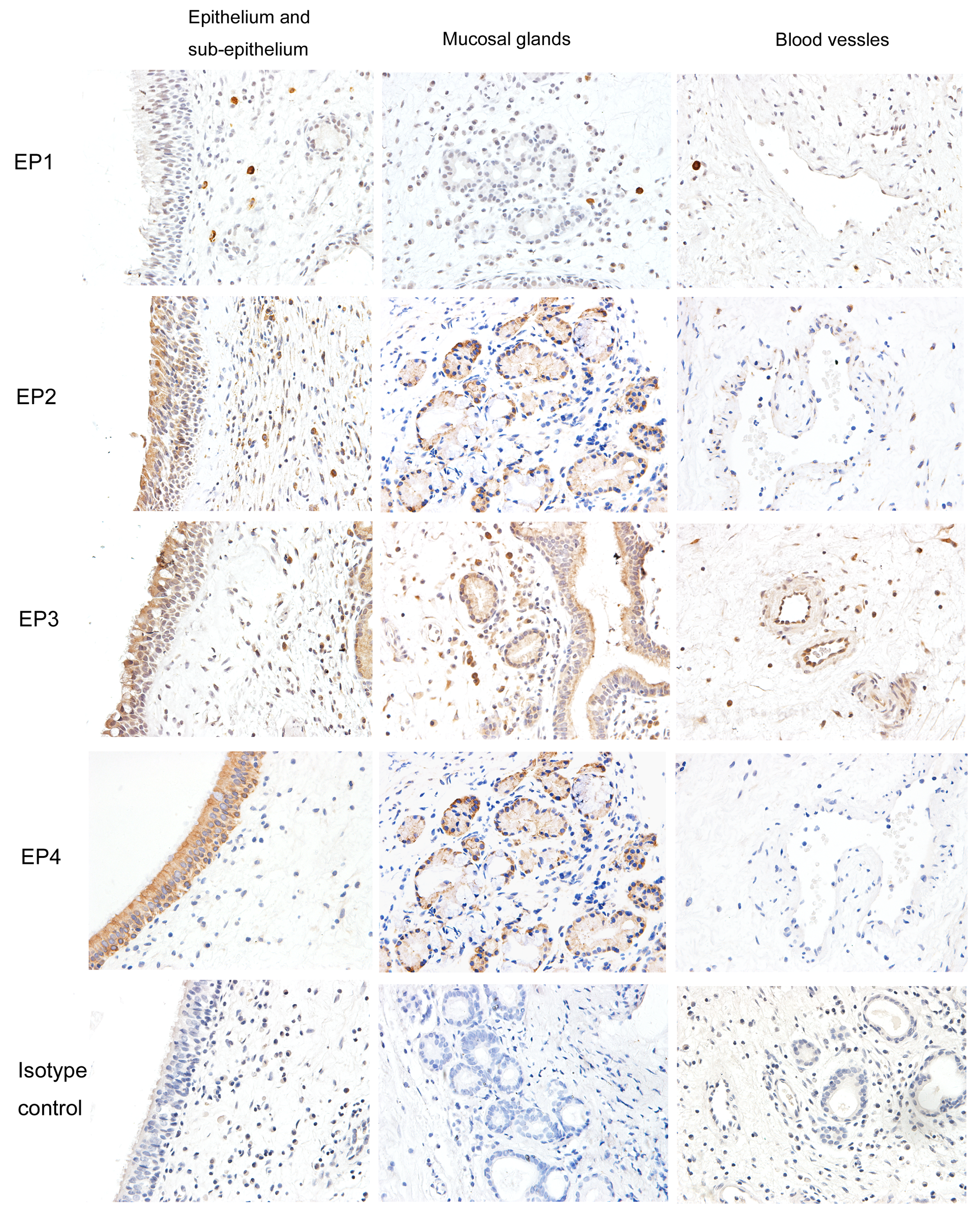

Supplement: S1 Fig — (TIF) [file pone.0200989.s001.tif]
